# Struggle in the bubble - a prospective study on the effect of remote learning and distance education on confidence in practical surgical skills acquired during COVID-19

**DOI:** 10.1186/s12909-023-04092-9

**Published:** 2023-02-15

**Authors:** Felicia Kneifel, Haluk Morgul, Shadi Katou, Jens P. Hölzen, Benjamin Strücker, Mazen Juratli, Andreas Pascher, Felix Becker

**Affiliations:** Department of General, Visceral and Transplant Surgery, University Hospital Münster, Münster, Germany

**Keywords:** Student education, Surgery, Basic surgical skills, Coronavirus disease

## Abstract

**Background:**

The coronavirus disease (COVID-19) has significantly changed healthcare systems and medical education. Universities were required to develop innovative curricula based on remote and distance education to continue medical education. This prospective questionnaire-based study aimed to investigate the impact of COVID-19-associated remote learning on the surgical training of medical students.

**Methods:**

A 16-item questionnaire-based survey was distributed to medical students at the University Hospital of Münster before and after a surgical skills laboratory (SSL). Two cohorts were included: summer semester 2021 (COV-19), with rigorous social-distancing restrictions requiered SSL to be remotely, and winter semester 2021 (postCOV-19), in which the SSL was provided as a face-to-face, hands-on course.

**Results:**

Both, cohorts showed a significant improvement in self-assessment of pre- and post-course confidence. While no significant difference in the average gain in self-confidence for sterile working was observed between the two cohorts, improvement in self-confidence was significantly higher in the COV-19 cohort regarding skin suturing and knot tying (*p* < 0.0001). However the average improvement regarding history and physical was significantly higher in the postCOV-19 cohort (*p* < 0.0001). In subgroup analysis, gender-associated differences varied in the two cohorts and were not related to specific subtasks, while age-stratified analysis revealed superior results for younger students.

**Conclusion:**

The results of our study underline the usability, feasibility, and adequacy of remote learning for the surgical training of medical students. The on-site distance education version, presented in the study, allows the continuing of hands-on experience in a safe environment in compliance with governmental social-distancing restrictions.

**Supplementary Information:**

The online version contains supplementary material available at 10.1186/s12909-023-04092-9.

## Background

The coronavirus disease (COVID-19) pandemic and subsequent mitigation strategies have unpredictably altered approximately every aspect of public life, particularly global healthcare systems and medical education [1]. As a result of the new social-distancing restrictions, medical education has been uniquely impaired. COVID-19 pandemic forced medical education faculty to a transition from pre-clerkship curriculum to online formats. In addition to the postponement of face-to-face teaching, hands-on courses were surceased, clinical skill courses were transferred online, and the students had to withdraw from in-hospital clerkships. Especially in medical students’ education, the challenge is providing authentic experiences and practical skills as a core feature of their curriculum. Since continuing education for medical students is crucial in maintaining the competency of healthcare professionals, innovative teaching methods were implemented [2–5]. Current literature has illuminated contradictory pros and cons referring to the impact of the COVID-19 pandemic on medical students’ education. Commonly proposed method for maintaining students’ education comprise scheduled live online video lectures (webinars) and interactive discussions. Besides this, various online platforms and programs enable self-studying of recorded lectures [6, 7]. A recent study illustrated a strong increase in the demand for online learning, with a 38% rise in the users of online teaching programs since the pandemic began [2]. Additionally, advocates emphasize the opportunity for increasing impact to reach more students by expanding learning opportunities through guest lecturers and access to subject matter experts [8, 9]. Notably, the scope of online teaching programs is primarily observational and has not been codified. While feasibility of online learning programs is obvious [4, 10, 11], several studies have investigated how the sudden shift to online learning in the context of COVID-19 affected students’ education. Altindag et al. elucidated the superiority of in-person compared to online courses showing data from a public university using a student fixed-effect model [12]. Moreover, a current publication by Kofoed et al. illustrated that performance of students randomly assigned to an online introductory economic class was lower than their peers who participated in in-person courses [13]. Furthermore, a recent German survey of medical students investigating the effects of e-learning for teaching surgical skills emphasized that practical surgical competencies cannot be adequately represented by online offers [14]. Notwithstanding, online teaching of basic surgical skills emerged to be effective and therefore shows potential to overcome the limits imposed by the COVID-19 crisis. However, there is limited evidence for online teaching of practical surgical skills, and the long-term repercussions of these training programs remain uncharted [15–17].

An established model for higher education institutes is blended learning, which combines in part supervised face-to-face classes on campus and in part online materials provided on the Internet. In contrast to traditional learning settings, blended learning offers self-directed learning and flexible routines for individual learning processes [18]. However, while the benefits of blended learning have been established for preclinical courses (e.g., anatomy) [19], the understanding regarding remote learning and distance education in clinical courses is still lacking. In this regard, a recent online, cross-sectional, national survey investigating online teaching during the COVID-19 pandemic demonstrated that clinical teaching received via direct patient contact was not successfully replaced by online teaching. In particular, practical clinical skills could not be imparted through remote learning, indicating that clinical skills were a potential barrier to online teaching and remote learning [20]. Therefore, medical schools worldwide were impelled to decide between arresting their educational curricula or devising novel adapted versions of disseminating knowledge and skills [21, 22]. This is challenging, particularly for surgical training, as a high level of tutor–student interaction is required to impart technical and professional skills.

Furthermore, halting surgical educational programs would substancially impact residents, medical students, and consequently, the global surgical community. Therefore, the Department of General Visceral and Transplant Surgery of University of Münster developed a new curriculum based on remote learning and distance education to continue medical and surgical education, as well as to provide an opportunity for continuous learning while avoiding delays due to the pandemic [23, 24]. Traditionally, medical–surgical education at the University of Münster has been taught in a surgical skills laboratory (SSL), which is organized and conducted by the Department of General, Visceral, and Transplant Surgery. This interactive SSL has been in effect for more than 10 years and was designed as a hands-on learning model consisting of three didactic units with 13 subunits. The three units include 1) Sterile working [surgical scrubbing in and donning of the sterile gowns and sterile gloves (assisted by scrub tech and alone) and sterile wound management]; 2) knot tying and skin suturing [forehand throw, backhand throw, simple interrupted stitch, vertical mattress suture (Donati), and subcuticular running stich]; 3) history and physical [structured history, physical and abdominal examination (appendicitis and cholecystitis)].

As face-to-face learning was disrupted due to the COVID-19 pandemic in the Spring of 2021, the SSL had to be modified to continue the surgical education of medical students. Hence, SSL training was performed considering social-distancing restrictions in small but spatially separated groups (social bubbles) to avoid in-person tutor–student interactions. However, surgical education poses an eminent challenge when considering distance and remote learning, given that there has not been an adequate alternative for mastering surgical techniques with hands-on experience [25]. Therefore, to ensure the integrity and continuity of surgical education, it is important to assess the usability of unprecedented learning methods and determine their feasibility and adequacy for medical students. The present prospective questionnaire-based study aimed to evaluate the impact of COVID-19-associated restrictions on the surgical education of medical students, exemplified by basic surgical skills acquired in an SSL, and compared face-to-face tutorial courses with remote learning.

## Materials and methods

### Study design and population

This study included a prospective, questionnaire-based survey of two cohorts of third-year medical students who undertook the SSL training at the University of Münster between 2021 [full COVID-19 restrictions (COV-19)] and 2022 [reduced COVID-19 restrictions (postCOV-19)]. A focused group of clinical teaching fellows and current Foundation year 1 (FY1) doctors from the Department of General, Visceral, and Transplant Surgery, University Hospital Münster, created a 16-item questionnaire, combining dichotomous multiple-choice and Likert scale-based questions. The questionnaire included demographic data (age and sex), as well as prior surgical experience.

**Fig. 1:**
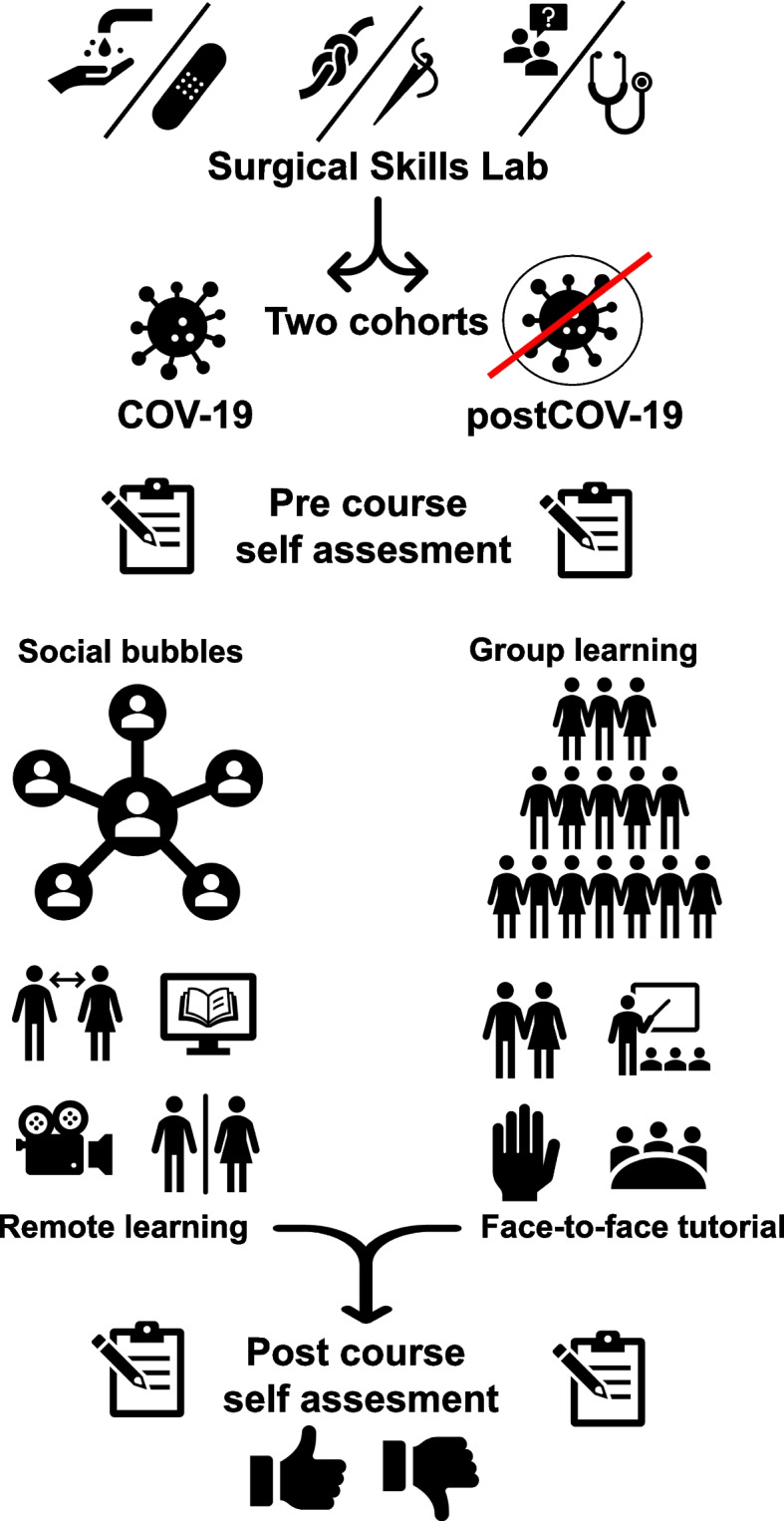
Study design. A prospective questionnaire-based study aiming to evaluate the impact of COVID-19-associated restrictions on medical students´ surgical education exemplified by basic surgical skills (sterile working, knot tying and skin suturing, history and physical) acquired in surgical skills laboratory and by comparing face-to-face tutorial courses with remote learning during social distancing

To assess the differences between both teaching models (in-person and remote education), the questionnaire was created to evaluate the self-confidence of students regarding basic surgical skills using a 6-point Likert scale of agree-ableness (0 = not confident at all; 1 = not very confident; 2 = neither completely confident nor underconfident; 3 = slightly confident; 4 = fairly confident; 5 = confident; 6 = completely confident) throughout the course. The anonymous and non-mandatory questionnaire (Supplementary Material) was administrated to the participating students before the first and after the last course of the SLL (Fig. 1).

### Social-distancing restrictions at the University of Münster

In addition to the implementation of a digital curriculum (online lectures and interactive webinars), the University of Münster divided the semester cohorts into social bubbles that comprised 4–6 students to enable the continuation of on-campus education. These social bubbles were encouraged not to come in physical contact with each other. Because of the governmental social-distancing restrictions and to protect the teachers and tutors, they were asked not to get in contact with the students in person.

### Changes in the SSL training during COVID-19

#### COV-19

For the COV-19 cohort, the SSL training was performed in social bubbles to adhere to the social-distancing restrictions. The course was performed in 12 separate exam rooms that were arranged around a central control room, bordered by glass panes. Each exam room was equipped with an examination couch and a computer to display teaching videos. The tutor inside the control room was able to communicate with the students (in a single room or all rooms) using a microphone. The SSL training was simultaneously performed with two social bubbles using different entrances to ensure that the students did not come in contact with each other during the training. Each social bubble was divided into groups of two students that shared one exam room. Following a short introductory presentation on the learning objectives of the current course, the students were assumed to perform the procedures by themselves. The teaching videos were provided for practical procedures (e.g. knot tying), and during the course, the students were able to get in contact with the tutor via a microphone.

#### PostCOV-19

For the post-COV-19 cohort, the SSL training was performed as a face-to-face tutorial. Each class consisted of 12–18 students, and the courses were conducted in a large seminar room. Following a short introductory lecture, the practical procedures were explained by the tutor. Afterwards, the students performed the procedures themselves with the support and supervision of the tutor.

### Statistical analyses

Data collection and statistical analyses were performed using Microsoft Excel 2010 (Microsoft Corporation, Redmond, WA, USA) and GraphPad Prism 9 for macOS version 9.3.1 (GraphPad Software, San Diego, CA, USA). Descriptive statistics were used to examine the characteristics and responses of participants using frequencies and percentages. Categorial variables were described as frequencies and percentages, and continuous variables as mean [± standard error of the mean (SEM)]. Moreover, categorical variables were compared using Fisher’s exact test, and continuous variables were compared using Student’s *t*-test. For comparison of more than two groups, one-way analysis of variance (ANOVA) with the Bonferroni post-hoc test was performed. A *p*-value < 0.05 was considered statistically significant.

## Results

### Demographics

A total of 232 out of 244 medical students completed both the baseline and follow-up questionnaire-based surveys, resulting in a response rate of 95%. Demographic data was comparable between the two cohorts (Table 1).

**Table 1:** Baseline comparison of the characteristics of participants belonging to the COV-19 and postCOV- 19 cohorts

		Total (*n* = 232)	*COV-19* (*n* = 112)	*PostCOV-19* (*n* = 120)	*p-value*
**Sex [n, (%)]**	Male Female	82 (35) 150 (65)	33 (29) 79 (71)	49 (41) 71 (59)	*0.0756*
**Age [years, n (%)]**	19–22 23–29 ≥ 30	146 (63) 64 (28) 19 (8)	76 (68) 25 (22) 11 (10)	70 (58) 39 (33) 8 (7)	*0.1592*
**Prior surgical** **experience [n, (%)]**	Yes No	69 (30) 163 (70)	34 (30) 78 (70)	35 (29) 85 (71)	*0.8862*

Data are presented as relative frequencies and compared using Fisher’s exact test

*COV-19* cohort of summer semester 2021 (with full COVID-19 restrictions), *postCOV-19* cohort of winter semester 2021/ 2022 (with reduced COVID-19 restrictions)

A *p*-value < 0.05 was considered statistically significant

#### Improvement in self-confidence for unit 1

**Fig. 2:**
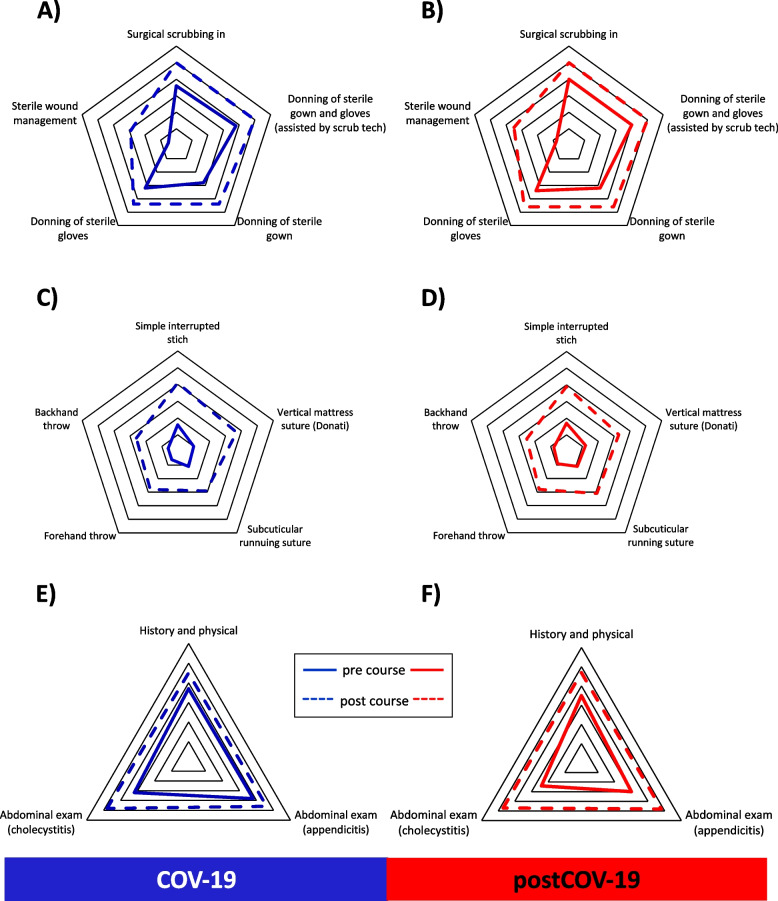
Self-assessment comparing pre- and post-course confidence of COV-19 and postCOV-19. Spider web graphs displaying the difference between pre- (full line) and post- (dotted line) course self-assessment. Unit 1 (sterile working): **A** (COV-19) + **B** (postCOV-19); unit 2 (knot tying and skin suturing): **C** (COV-19) + **D** (postCOV-19); unit 3 (history and physical): **E** (COV-19) + **F** (postCOV-19). COV-19 = cohort of summer semester 2021 (full COVID-19 restrictions), postCOV-19 = cohort of winter semester 2021/2022 (reduced COVID-19 restrictions)

First, it was evaluated whether the respective teaching methods in both cohorts resulted in an improvement in the self-confidence of students regarding their surgical skills. While analyzing unit 1 (sterile working), we found that both the COV-19 (Fig. 2A) and postCOV-19 (Fig. 2B) cohorts showed significant improvement in post-course confidence compared to pre-course confidence. This result was observed for all five subcategories of unit 1 (Table 2).

**Table 2:** Self-assessment of pre- and post-course confidence of unit 1

	*COV-19* (*n* = 112)			*PostCOV-19* (*n* = 120)		
	***Self-assessment*** (mean ± SEM)					
	Pre- course	Post- course	***p***-value	Pre- course	Post-course	***p***-value
Surgical scrubbing	3.58 ± 0.21	5.03 ± 0.09	*< 0.0001*	3.95 ± 0.17	5 ± 0.12	*< 0.0001*
Donning of sterile gown and gloves (assisted by scrub tech)	3.82 ± 0.19	5.02 ± 0.09	*< 0.0001*	3.99 ± 0.17	4.97 ± 0.11	*< 0.0001*
Donning sterile gown	2.76 ± 0.19	4.43 ± 0.11	*< 0.0001*	3.17 ± 0.17	4.59 ± 0.13	*< 0.0001*
Donning sterile gloves	3.22 ± 0.17	4.38 ± 0.12	*< 0.0001*	3.4 ± 0.16	4.56 ± 0.12	*< 0.0001*
Sterile wound management	0.46 ± 0.11	2.87 ± 0.16	*< 0.0001*	0.78 ± 0.14	3.47 ± 0.17	*< 0.0001*

Data are presented as mean ± SEM and compared using Student’s *t*-test

*COV-19* cohort of summer semester 2021 (with full COVID-19 restrictions), *postCOV-19* cohort of winter semester 2021/ 2022 (with reduced COVID-19 restrictions), *SEM* standard error of the mean

A *p*-value < 0.05 was considered statistically significant

#### Improvement in self-confidence for unit 2

While analyzing unit 2 (knot tying and skin suturing), we observed that both the COV-19 (Fig. 2C) and postCOV-19 (Fig. 2D) cohorts exhibited significant improvement in post-course confidence compared to pre-course confidence. This result was similar for all five subcategories of unit 2 (Table 3).

**Table 3:** Self-assessment of pre- and post-course confidence of unit 2

	*COV-19* (*n* = 112)			*PostCOV-19* (*n* = 120)		
	***Self-assessment*** (mean ± SEM)					
	Pre-course	Post-course	***p-***value	Pre-course	Post-course	***p-***value
Simple interrupted stich	1.59 ± 0.21	4.13 ± 0.16	*< 0.0001*	1.70 ± 0.19	3.91 ± 0.13	*< 0.0001*
Vertical mattress suture (Donati)	1.01 ± 0.18	3.69 ± 0.17	*< 0.0001*	1.21 ± 0.16	3.31 ± 0.14	*< 0.0001*
Subcuticular running suture	1.08 ± 0.13	2.91 ± 0.18	*< 0.0001*	1.10 ± 0.14	3.08 ± 0.14	*< 0.0001*
Forehand throw	0.62 ± 0.12	2.80 ± 0.17	*< 0.0001*	0.90 ± 0.13	2.79 ± 0.16	*< 0.0001*
Backhand throw	0.58 ± 0.11	2.63 ± 0.17	*< 0.0001*	0.80 ± 0.13	2.50 ± 0.16	*< 0.0001*

Data are presented as mean ± SEM and compared using Student’s *t*-test

*COV-19* Cohort of summer semester 2021 (with full COVID-19 restrictions), *postCOV-19* cohort of winter semester 2021/ 2022 (with reduced COVID-19 restrictions), *SEM* standard error of the mean

A *p*-value < 0.05 was considered statistically significant

#### Improvement in self-confidence for unit 3

Upon analyzing unit 3 (history and physical), we identified that both, the COV-19 (Fig. 2E) and postCOV-19 (Fig. 2F) cohorts, revealed significant improvement in post-course confidence compared to pre-course confidence. This result was observed for all three subcategories of unit 3 (Table 4).

**Table 4:** Self-assessment of pre- and post-course confidence of unit 3

	*COV-19* (*n* = 112)			*PostCOV-19* (*n* = 120)		
	***Self-assessment*** (mean ± SEM)					
	Pre-course	Post-course	***p-***value	Pre-course	Post-course	***p-***value
History and physical	3.71 ± 0.13	4.50 ± 0.12	*< 0.0001*	3.53 ± 0.13	4.68 ± 0.10	*< 0.0001*
Abdominal exam (appendicitis)	3.75 ± 0.13	4.61 ± 0.13	< *0.0001*	3.00 ± 0.13	4.78 ± 0.09	*< 0.0001*
Abdominal exam (cholecystitis)	3.16 ± 0.15	4.81 ± 0.10	*< 0.0001*	2.35 ± 0.13	4.70 ± 0.09	*< 0.0001*

Data are presented as mean ± SEM and compared using Student’s *t*-test

*COV-19* cohort of summer semester 2021 (with full COVID-19 restrictions), *postCOV-19* cohort of winter semester 2021/ 2022 (with reduced COVID-19 restrictions), *SEM* standard error of the mean

A *p*-value < 0.05 was considered statistically significant

Having established that both the traditional interactive face-to-face hands-on courses and the newly developed interactive remote learning courses were able to significantly improve the confidence of medical students regarding basic surgical skills, it was necessary to determine the course that resulted in a higher difference between the pre- and post-course confidence and the subgroup of students that would benefit the most from a particular teaching method. Subgroup analysis was performed based on sex (male/female), age group (19–22 years/23–29 years/≥30 years), and prior surgical experience (with and without prior surgical experience) for evaluating the difference between the pre- and post-course self-assessment (Δ self-assessment).

### Subgroup analysis

#### Sex

**Fig. 3:**
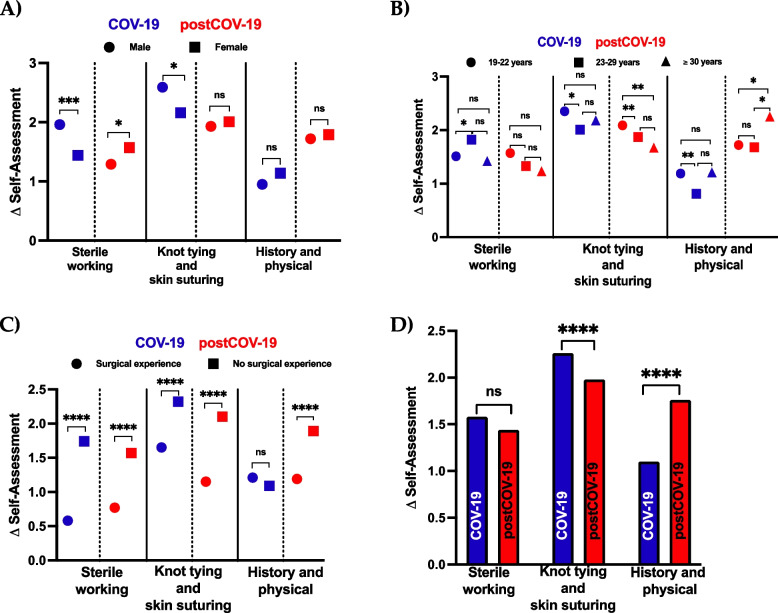
Subgroup analysis comparing pre- and post-course self-assessment (Δ self-assessment). **A** subgroup (sex: male vs. female) analysis for differences in Δ self-assessment, **B**) subgroup (age: 19–22 years vs. 23–29 years vs. ≥ 30 years) analysis for differences in Δ self-assessment, **C**) subgroup (prior surgical experience: with vs. without surgical experience) analysis for differences in Δ self-assessment, **D**) analysis for differences in Δ self-assessment comparing COV-19 vs. postCOV-19. Data are presented as mean and compared using Student’s t-test or ANOVA. A *p*-value less than 0.05 was considered statistically significant. Significance is indicated by the following symbols: * *p* < 0.05, ** *p* < 0.01, *** *p* < 0.001, **** *p* < 0.00001, ns = not significant. COV-19 = cohort of summer semester 2021 (full COVID-19 restrictions), postCOV-19 = cohort of winter semester 2021/2022 (reduced COVID-19 restrictions)

The cohorts were first stratified based on the sex (male or female) of the participants, and the subgroup that benefited the most from a particular learning method was determined. For unit 1, the mean Δ self-assessment in the COV-19 cohort was significantly higher in male students (1.96) than in female students (1.44) (*p* = 0.0003). However, in the postCOV-19 cohort, the mean Δ self-assessment was significantly higher in female students (1.57) compared to male students (1.29) (*p* = 0.0372) (Fig. 3A).

For unit 2, the mean Δ self-assessment in the COV-19 cohort was significantly higher in male students (2.59) compared to female students (2.16) (*p* < 0.0001), whereas no significant difference between males (1.92) and females (2.01) was observed in the mean Δ self-assessment in the postCOV-19 cohort (*p* = 0.0813) (Fig. 3A).

Nonetheless, for unit 3, we found that the mean Δ self-assessment was comparable between the female and male groups in both cohorts (Fig. 3A).

#### Age

The two cohorts were stratified based on age, which resulted in three subgroups: 19–22, 23–29, and ≥ 30 years. For unit 1, we found that the mean Δ self-assessment in the COV-19 cohort was the highest for the participants in the age group of 23–29 years (mean Δ self-assessment = 19–22 years: 1.51; 23–29 years: 1.82; ≥30 years: 1.42). Furthermore, the mean Δ self-assessment was significantly higher in students of ages 23–29 years compared to those in the age group of 19–22 years (*p* = 0.0234). However, no significant differences in the mean Δ self-assessment were observed between the subgroups 19–22 years and ≥ 30 years (*p* = 0.8443), as well as the subgroups 23–29 years and ≥ 30 years (*p* = 0.0761).

By contrast, the mean Δ self-assessment of unit 1 did not vary significantly between different age groups in the postCOV-19 (mean Δ self-assessment = 19–22 years: 1.58; 23–29 years: 1.33; ≥30 years: 1.23) cohort (Fig. 3B).

Considering unit 2, we determined that the youngest (19–22 years) subgroup exhibited the maximum improvement in self-assessment for the COV-19 and post-COV19 cohorts. In the COV-19 cohort, the mean Δ self-assessment was significantly higher in the subgroup with participants aged 19–22 years compared to the subgroup with participants aged 23–29 years (*p* = 0.0017). However, there was no significant difference between the subgroups with participants aged 19–22 years and ≥ 30 years (*p* = 0.4096), as well as the subgroups with participants aged 23–29 years and ≥ 30 years (*p* = 0.5073).

In the postCOV-19 cohort, the mean Δ self-assessment was significantly higher in the subgroup with participants aged 19–22 years compared to the subgroups with participants aged 23–29 years (*p* = 0.0020) and ≥ 30 years (*p* = 0.0017). In contrast, there was no significant difference observed between the mean Δ self-assessment of the subgroups with participants aged 23–29 years and ≥ 30 years (*p* = 0.2499) (Fig. 3B).

Upon analyzing unit 3, the mean Δ self-assessment in the COV-19 cohort was significantly higher in the youngest students (19–22 years) compared to the subgroup with participants aged 23–29 years (*p* = 0.0061) in COV-19. However, there was no significant difference in the mean Δ self-assessment between the participants aged 19–22 years and ≥ 30 years (*p* = 0.0934) and 23–29 years and ≥ 30 years (*p* = 0.9923).

Nonetheless, for unit 3, the mean Δ self-assessment was significantly higher in the subgroup with participants aged ≥30 years compared to subgroups with participants aged 19–22 years (*p* = 0.0224) and 23–29 years (*p* = 0.0181) in the postCOV-19 cohort (mean Δ self-assessment = 19–22 years: 1.73; 23–29 years: 1.68; ≥30 years: 2.35). However, no significant difference was noted in the mean Δ self-assessment of subgroups with students aged 19–22 years and 23–29 years (*p* = 0.9332) in the postCOV-19 cohort (Fig. 3B).

#### Prior surgical experience

Lastly, the two cohorts were stratified based on prior surgical experience. Students without prior surgical experience showed a significantly higher improvement in their self-assessment of post-course confidence compared to pre-course confidence. This result was found for unit 1 and 2 in the COV-19 (unit 1 = mean Δ self-assessment with surgical experience: 0.58; without surgical experience: 1.74; *p* < 0.0001; unit 2 = mean Δ self-assessment with surgical experience: 1.65; without surgical experience: 2.14; *p* < 0.0001) and postCOV-19 cohorts (unit 1 = mean Δ self-assessment with surgical experience: 0.77; without surgical experience: 1.57; *p* < 0.0001; unit 2 = mean Δ self-assessment with surgical experience: 1.15; without surgical experience: 2.10; *p* < 0.0001).

However, for unit 3, we observed that the mean Δ self-assessment did not vary significantly between students with and without prior surgical experience in the COV-19 cohort (mean Δ self-assessment with surgical experience: 1.21; without surgical experience: 1.09; *p* = 0.2242) but was significantly higher for students without surgical experience in the postCOV-19 cohort (mean Δ self-assessment with surgical experience: 1.19; without surgical experience: 1.89; *p* < 0.0001) (Fig. 3C).

To summarize, the mean Δ self-assessment was the highest in the young (19–22 years) male students without surgical experience in the COV-19 cohort and young (19–22 years) and elderly (≥30 years) female students without surgical experience in the postCOV-19 cohort.

Finally, we compared the mean Δ self-assessment of both cohorts using each unit. Both, the COV-19 (Δ self-assessment: 1.58) and postCOV-19 (Δ self-assessment: 1.46) cohorts showed comparable (*p* = 0.1485) results for unit 1. For unit 2, the mean Δ self-assessment was significantly (*p* < 0.0001) higher in the COV-19 cohort (Δ self-assessment: 2.26) compared to the postCOV-19 (Δ self-assessment: 1.98). In contrast, for unit 3, the Δ self-assessment was significantly (*p* < 0.0001) higher in the postCOV-19 cohort (Δ self-assessment: 1.76) compared to the COV-19 cohort (Δ self-assessment: 1.1) (Fig. 3D).

## Discussion

This questionnaire-based study was designed to evaluate the impact of COVID-19-associated changes on the surgical education of medical students by evaluating basic surgical skills acquired during face-to-face tutorials compared to those acquired through remote learning. We hypothesized that on-site distance learning was comparable to face-to-face hands-on courses in teaching practical surgical skills in the context of SSL. The study demonstrated that social distancing was not an obstacle in teaching basic surgical skills to medical students.

Medical education worldwide has been severely affected by the COVID-19 pandemic [1, 26]. However, this cross-border crisis has provided an unprecedented stimulus for educational novelties. Accordingly, COVID-19 has called for a rapid adaptation of medical education and has forced higher education institutes to switch to virtual platforms, online and blended learning. Medical educators have been and are still confronted with substantial challenges as the established models, such as classroom-based, face-to-face teaching, have been disrupted due to social-distancing restrictions [27]. Notwithstanding, online communication platforms have provided an important approach to continue medical education. While lectures or interactive seminars can be easily delivered online, it has been challenging to conduct surgical training (requiring a high level of teacher–student interactions) online or via pre-recorded videos [14–17, 28]. Although, scattered studies reported feasibility of online teaching for practical surgical skills, video-based education constitutes a passive learning mode and will not be able to substitute hands-on courses [15–17]. Nevertheless, the long-term impact of the integration of virtual learning, especially for teaching of practical skills remains unknown and must be analyzed for effectiveness. Besides, online education and assessment constitute its own challenges. First, dependability of online assessment systems, especially network connectivity needs to be stable to be used reliantly. Second, students may lack a home environment appropriate to attend a sitting or examination or may have obstacles to access adequate online facilities [29]. Further, the role of teacher–student interactions in the context of media-based education of practical surgical skills is under controversial discussion [30–32].

Undoubtedly, the COVID-19 pandemic is still going on and therefore will continue to disrupt students’ medical and surgical education and training. As we are facing the fourth and fifth wave of this pandemic in Germany, several measures must be implemented to minimize the impact on medical students’ surgical education.

However, while practical surgical skills cannot be transferred over remote learning, steps can be taken to ensure maximal clinical exposure. Thus, our institution implemented an adapted, non-contact, on-site version of surgical skills training for the medical students to overcome two of the major obstacles faced in medical education during COVID-19 — the lack of practical education and the difficulty in receiving real-time feedback from a tutor.

In our institute, the SSL training was delivered through real-time skill demonstration via a camera. The students were allowed to ask questions and were asked to demonstrate surgical tasks while obtaining real-time feedback, clues, and adjustments. In addition, we provided access to online teaching videos to overcome the lack of hands-on experience.

This study suggests that there was no significant difference in the average gain in self-confidence for basic surgery skills, such as surgical scrubbing in, gowning, gloving, and working in a sterile field, acquired during the SSL training, between interactive, face-to-face hands-on courses and interactive, remote learning courses. Moreover, the average improvement in knot tying and skin suturing was higher in the COV-19 cohort. This can be attributed to the ability of explicitly demonstrating surgical skills via camera which allowed equal conditions for all students. In contrast, face-to-face tutorials in large groups involved limited viewing for students sitting at the back of the seminar room. Nevertheless, improvement in the history and physical unit was superior in the postCOV-19 cohort. Accordingly, imparting competencies in physical examination via media-based learning was also challenging as it required a high level of teacher–student interaction. Thus, teaching examination methods through remote learning and distance education might necessitate further conceptions and amelioration.

In the subgroup analysis, we identified the course that resulted in an increased difference between the pre- and post-course evaluations and the subgroup of students that benefitted the most from a particular teaching method. Subgroup analysis indicated significant differences in the pre- and post-course self-assessments for both the COV-19 and postCOV-19 cohorts, based on gender and age of the participants. The gender-associated differences, however, varied in the two cohorts and were not related to specific subtasks. Although the subgroup analysis of the three different age groups indicated higher learning ability in the age groups of 19–22 years and 23–29 years, particularly for the subtasks of units 1 and 2 for both, the COV-19 and postCOV-19 cohorts, there was no distinct superiority inferable for a specific age group. Nevertheless, the mean Δ self-assessment was significantly higher in the subgroups without prior surgical experience. This result was consistent for all subcategories except for unit 3 in the COV-19 cohort.

There were several limitations in this study. First, it was performed in a single country and in a single university, comprising a homogenous group of participants. Moreover, the sample size was relatively small. Further, this study is based on students’ self-assessed confidence with sets of skills rather than on an objective assessment of their actual competence in these skills. Thus, the main outcome measure was not a validated objective measure. Therefore, we did not perform general linear models. However, our findings may not be generalized to other Universities or other countries and must be interpreted with caution. Given these limitations, future research evaluating the impact of distance education and blended learning models in teaching of practical surgical skills is needed. Replication studies in variable contexts could be helpful to determine whether distance learning is able to replace the traditional, face-to-face hands-on courses and thus providing a suitable solution for the disruption of clinical training caused by COVID-19 pandemic. Intending to objectively assess the efficiency of distance teaching of basic surgical skills, further studies need to use validated measurable outcomes.

However, the presented adapted version of the SSL training was affordable as it did not necessitate a highly specialized software program or advanced equipment, except for a camera setup and a video conferencing platform, to ensure surgical education of medical students, particularly during challenging time of COVID-19 pandemic. In addition, this on-site version of distance education circumvents obvious drawbacks of online teaching [15, 29], as the essential equipment is provided by the academic institution and there is no need of appropriate home environment or internet/ network access for participating students.

## Conclusions

The average improvement in self-confidence was comparable between the COV-19 and postCOV-19 cohorts for sterile working. However, improvement in self-confidence regarding skin suturing and knot tying was significantly higher in the COV-19 cohort, whereas the average improvement in self-confidence regarding the history and physical unit was significantly higher in the postCOV-19 cohort. The findings of this study underline the usability, feasibility, and adequacy of remote learning methods during social-distancing restrictions for the practical surgical education of medical students. Due to the ongoing COVID-19 crisis, there is an unmet need of valid solutions aiming to reduce the disruption of medical students’ surgical education and thus minimizing the impact on medical education and the progression of training. The adapted version, presented in the study, of on-site distance education allows the continuing of hands-on experience in a safe environment, in compliance with governmental social-distancing restrictions.

## Supplementary Information

**Additional file 1.**

## Data Availability

The datasets used and/or analyzed during the current study are available from the corresponding author on reasonable request.

## Abbreviations

COVID-19: Coronavirus disease
SSL: Surgical skills lab
COV-19: Cohort of summer semester 2021 (full COVID-19 restrictions)
postCOV-19: Cohort of winter semester 2021/2022 (reduced COVID-19 restrictions)
FY1: Foundation year 1
