# Supplementary material for: Struggle in the bubble - a prospective study on the effect of remote learning and distance education on confidence in practical surgical skills acquired during COVID-19
Source: BMC Med Educ. 2023 Feb 15;23:115. doi: 10.1186/s12909-023-04092-9 (PMC9931444; doi:10.1186/s12909-023-04092-9)
Supplement: Supplementary file 1 [file 12909_2023_4092_MOESM1_ESM.docx]

Struggle in the Bubble - A Prospective Study on the Effect of Remote Learning and Distance Education on Confidence in Practical Surgical Skills Acquired during COVID-19

Questionnaire for third-year medical students (Surgical Skills Laboratory):

Age: ___________ *Gender: male / female*

(Please tick as appropriate)

1) Have you completed an internship in general and visceral surgery?

2) If yes, how long?

1-2 weeks 2-4 weeks >4 weeks

3) Do you have a student job in a surgical specialty?

Please answer the following questions and assign a score between 0-6 for each:

0 = not confident at all; 1 = not very confident; 2 = neither completely confident nor underconfident; 3 = slightly confident; 4 = fairly confident; 5 = confident; 6 = completely confident

1) Do you feel confident to scrub in under the supervision of a scrub tech?

0 1 2 3 4 5 6

2) Do you feel that you can don a sterile gown and gloves assisted by scrub tech?

0 1 2 3 4 5 6

3) Do you feel able to don a sterile gown by yourself under the supervision of a scrub tech?

0 1 2 3 4 5 6

4) Do you feel able to don sterile gloves by yourself under the supervision of a scrub tech?

0 1 2 3 4 5 6

5) Do you feel able to fix abdominal drainage by forehand throw?

0 1 2 3 4 5 6

6) Do you feel able to fix abdominal drainage by backhand throw?

0 1 2 3 4 5 6

7) Do you feel able to do an epidermal closure by simple interrupted stitch?

0 1 2 3 4 5 6

8) Do you feel able to perform an epidermal closure by vertical mattress suture (Donati)?

0 1 2 3 4 5 6

9) Do you feel able to perform an epidermal closure by subcuticular running stitch?

0 1 2 3 4 5 6

10) Do you feel able to apply an epidermal wound VAC ®?

0 1 2 3 4 5 6

11) Do you feel able to take a structured and complete medical history and physical?

0 1 2 3 4 5 6

12) Do you feel able to perform an abdominal exam for appendicitis?

0 1 2 3 4 5 6

13) Do you feel able to perform an abdominal exam for cholecystitis?

0 1 2 3 4 5 6
